# Seed fungal endophytes as biostimulants and biocontrol agents to improve seed performance

**DOI:** 10.3389/fpls.2023.1260292

**Published:** 2023-10-24

**Authors:** Félix Rétif, Caroline Kunz, Kevin Calabro, Clémence Duval, Soizic Prado, Christophe Bailly, Emmanuel Baudouin

**Affiliations:** ^1^ Sorbonne Université, CNRS UMR7622, Institut de Biologie Paris-Seine-Laboratoire de Biologie du Développement (IBPS-LBD), Paris, France; ^2^ Muséum National d’Histoire Naturelle, Unité Molécules de Communication et Adaptation des Micro-organismes, UMR 7245, Paris, France; ^3^ Sorbonne Université, Faculté des Sciences et Ingénierie, UFR 927, Paris, France; ^4^ Seedlab, Novalliance, Zone Anjou Actiparc, Longué-Jumelles, France

**Keywords:** seeds, fungal endophytes, germination, stress tolerance, bio-stimulant, biocontrol, fungal metabolites

## Abstract

Seed germination is a major determinant of plant development and final yield establishment but strongly reliant on the plant’s abiotic and biotic environment. In the context of global climate change, classical approaches to improve seed germination under challenging environments through selection and use of synthetic pesticides reached their limits. A currently underexplored way is to exploit the beneficial impact of the microorganisms associated with plants. Among plant microbiota, endophytes, which are micro-organisms living inside host plant tissues without causing any visible symptoms, are promising candidates for improving plant fitness. They possibly establish a mutualistic relationship with their host, leading to enhanced plant yield and improved tolerance to abiotic threats and pathogen attacks. The current view is that such beneficial association relies on chemical mediations using the large variety of molecules produced by endophytes. In contrast to leaf and root endophytes, seed-borne fungal endophytes have been poorly studied although they constitute the early-life plant microbiota. Moreover, seed-borne fungal microbiota and its metabolites appear as a pertinent lever for seed quality improvement. This review summarizes the recent advances in the identification of seed fungal endophytes and metabolites and their benefits for seed biology, especially under stress. It also addresses the mechanisms underlying fungal effects on seed physiology and their potential use to improve crop seed performance.’

## Introduction

Endophytic microorganisms are part of the plant microbiome and reside transiently or permanently within plant tissues without causing disease symptoms (Wilson, 1995). Endophytic fungi mainly belong to the Ascomycota or Basidiomycota (Rashmi, 2019) and, together with bacteria, constitute the most abundant, diverse and ubiquitous group of endophytes. Their association with plants is attested since Devonian (Krings et al., 2007) and they have been detected in most plants studied so far and in a variety of plant organs including leaves, stems, roots, flowers, fruits and seeds (Stone et al., 2004). The classification of endophytic fungi integrates their phylogeny, their host range, extent of tissue colonization and transmission mode (Rodriguez et al., 2009). Their transmission is particularly important for the building of endophytic communities and their maintenance over space and time. Indeed, horizontal transmission assures constant supply of endophytes for (re)inoculation from plant environment whereas vertical transmission allows the transfer of endophytic populations from mother plants to their progeny via the seeds and possibly the maintenance of endophytic microbiome composition across generations (Bright and Bulgheresi, 2010; Abdelfattah et al., 2022).

A particular attention has been paid to endophytic fungi considering the numerous services they can provide to plants and their high potential for application in agriculture. Indeed abundant literature reports the capacity of fungal endophytes to promote plant growth and to improve their tolerance towards abiotic and biotic stresses, in exchange of nutriment supply and shelter (Baron and Rigobelo, 2022; Verma et al., 2022). As for seed-borne endophytic fungi, evidence also points to their ability to promote seed germination and early seedling growth, and possibly impact the whole plant development and response to environmental cues (Li et al., 2019). Interestingly, the positive effects of fungal endophytes largely rely on bioactive molecules they produce and that stimulate plant growth, and participate in adaptive responses or immunity (Prado et al., 2012; Lugtenberg et al., 2016). Indeed, the chemical repertoire of fungal endophytes is not only exceptionally diverse in itself, but also shaped by their environment within the plant, which makes these fungi a unique reservoir of bioactive molecules.

Seeds are unique structures found in Gymnosperms and Angiosperms that ensure their sustainability and dissemination, which is critical for species survival and spreading in ecosystems. They also constitute the income and outcome of major crop productions, and seed performance, including high germination rate, vigor or longevity, is crucial for plantlet establishment and final yield (Ellis, 1992). Due to their sensitivity towards environmental stresses, seed germination and possibly other seed traits are strongly jeopardized by ongoing climate change (Finch-Savage and Bassel, 2016). Moreover, global warming will enhance plant diseases, lowering yields and impairing food safety (Raza and Bebber, 2022). Because of on-going environmental policies and increasing concern for sustainable development, environmental-safe strategies to improve seed performance and resistance to pathogens are urgently required. In this view, the valorization of bio-sourced molecules as biostimulants, i.e. to promote plant growth and plant stress tolerance, or for the biocontrol of pathogens, is a promising strategy. Fungal endophytes, especially those naturally hosted in seeds, therefore appear as a valuable and original source of bioactive molecules. This review will bring an update on seed-borne fungal endophytes and their potential valorization to improve seed traits, i.e. dormancy, germination, vigor and longevity, under optimal or stress conditions, and seed tolerance towards pathogens and pests.

## Seed-borne endophytic fungi: population diversity and dynamics

Embodying bridges between successive generations, seeds have a central role in the conservation and transmission of the endophytic microbiome to the next generation. Recent studies have described the microbiome of crop and non-crop seeds using metagenomics approaches (Wassermann et al., 2019; Bintarti et al., 2022; Simonin et al., 2022). They highlight a high proportion of fungi in seed microbiota compared to other tissues, possibly reflecting the high capacity of fungal species to adapt to seed environment (Simonin et al., 2022). They also point out a strong diversity among plant species, between seeds of the same plant and even within the same fruit (Bintarti et al., 2022; Simonin et al., 2022). Indeed, seed endophytic fungal communities are not only dependent on host genetics, but are further shaped by environmental factors (Klaedtke et al., 2016; Wassermann et al., 2019; Franić et al., 2020; Bintarti et al., 2022; Philpott et al., 2023). Despite this variability, a handful of genera, *e.g. Alternaria, Phoma*, *Cladosporium*, *Fusarium*, *Xylaria*, *Penicillium* or *Aspergillus*, that are abundant and ubiquitously found in crops and wild species, emerge as the core endophytic community of seeds (Samreen et al., 2021; Simonin et al., 2022). In addition to these, *Epichloe* genus is widely present among *Poacea* (grass) seeds (Stone et al., 2004). Beside this core population, a diversity of additional endophytic fungal species have also been detected in seeds, that represent a minority and are highly flexible among plant species (Kluger et al., 2008; Klaedtke et al., 2016; Billingsley Tobias et al., 2017; Hill et al., 2021; Mertin et al., 2022; Simonin et al., 2022). Endophytic fungi from both core and flexible populations can improve seed performance (**Table 1**), which leaves open the relative contribution and functions of these fungal subgroups.

**Table 1:** Seed fungal endophytes showing bio-stimulant and/or biocontrol properties towards seeds.

Seed endophytic fungi	Host plant	Effect on seed biology	Possible mode of action	Reference
*Biostimulation*				
*Epichloë* sp.	*Achnatherum inebrians*	Improve seed dormancy release	Unknown	Chen et al., 2021
*Epichloë bromicola*	*Hordeum brevisubulatum*	Improve seed germination rate under salt stress	Unknown	Wang et al., 2020
*Epichloë festucae*	*Lolium perenne*	Improve seed germination rate	Unknown	Ma et al., 2015
*Epichloe gansuensis*	*Achnatherum inebrians*	Improve seed germination rate under salt, pH and temperature stress and various conditions of light	Unknown	Ahmad et al., 2020
*Epichloe gansuensis*	*Achnatherum inebrians*	Improve seed longevity	Higher peroxidase, superoxide dismutase and catalase activity Higher soluble sugar and proline content	Li et al;, 2020
*Epichloe gansuensis*	*Achnatherum inebrians*	Improve seed germination rate under sub-optimal temperature	Increased alkaloid biosynthesis, Upregulation of fatty acid biosynthesis, Upregulation of stress response molecules, Regulation of protein content	Chen et al., 2016
*Epichloë inebrians*	*Achnatherum inebrians*	Improve seed germination rate under wilder temperature range	Unknown	Bao et al., 2019
*Epichloë (Neotyphodium* sp.*)*	*Elymus dahuricus*	Improve seed germination rate germination improvement under cadmium stress	Higher peroxidase, ascorbate peroxidase, superoxide dismutase and catalase activity Higher proline content	Zhang et al., 2012
*Neotyphodium gansuense*	*Achnatherum inebrians*	Improve seed germination rate germination improvement under cadmium stress	Higher peroxidase, ascorbate peroxidase, superoxide dismutase and catalase activity Higher proline content	Zhang et al., 2010
*Acremonium coenophialum*	*Festuca arundinacea*	Improve seed number and weight	Unknown	Rice et al., 1990
*Acremonium loliae* *Acremonium coenophialum*	*Lolium perenne* *Festuca arundinacea*	Improve seed germination rate	Unknown	Clay, 1987
*Cladosporium cladosporioides*	*Suaeda salsa*	Improve seed germination rate	Unknown	Qin et al., 2016
*Epicoccum nigrum*	*Dysphania ambrosioides*	Increase seed production under cadmium stress	Auxin, gibberellin and jasmonic acid production by the endophyte Upregulation of the reduced glutathione content	Parmar et al., 2022
*Fusarium oxysporum* *Fusarium solani* *Fusarium* sp.	*Senna Alata*	Improve seed germination rate	Unknown	Pradhan et al., 2023
*Fusarium verticillioides*	*Glycine max*	Improve seed germination rate under salt stress	Higher protein content Lower ABA content	Radhakrishnan et al., 2013
*Penicillium* sp.	*Triticum turgidum*	Improve seed germination rate under heat and drought stress	Unknown	Hubbard et al., 2012
*Penicillium* sp.	*Triticum turgidum*	Increase seed number and weight under heat and drought stress during seed development and their germination	Unknown	Hubbard et al., 2014
*Penicillium* sp.	*Phragmites australis*	Improve seed germination rate	Unknown	Shearin et al., 2018
*Penicillium* sp.	*Triticum durum*	Improve seed dormancy release	Up-regulation of seed gibberellin signaling pathway	Vujanovic et al., 2016
***Biocontrol***				
*Epichloë* sp.	*Elymus sibiricus*	Improve seed germination rate under *Alternaria alternata, Bipolaris sorokiniana*, *Fusarium avenaceum*, and *Fusarium* sp. infections	Unknown	Li et al., 2017
*Epichloë festucae*	*Lolium perenne*	Improve seed germination rate under *Alternaria alternata*, *Ascochyta leptospora*, *Bipolaris sorokiniana*, *Curvularia lunata* and *Fusarium avenaceum* infections	Unknown	Ma et al., 2015
*Neotyphodium gansuense*	*Achnatherum inebrians*	Seed-harvesting ant deterence	Unknown	Zhang et al., 2011
*Acremonium ceoenophialum*	*Festuea arundinaeea*	*Pogonomyrmex rugosus* deterence	Unknown	Knoch et al., 1993
*Acremonium coenophialum*	*Festuca arundinacea*	*Junco hyernalis, Spizella arborea*, *Melospiza melodia* and *Passer domesticus* deterence	Unknown	Madej and Clay, 1991
*Acremonium loliae* *Acremonium coenophialum*	*Lolium perenne* *Festuca arundinacea*	Fall armyworm (*Spodoptera frugiperda*) and flour beetles (*Tribolium castaneum*) deterence	Ergot alkaloïd production	Cheplick and Clay, 1988

When known, the underlying mechanisms are indicated.

**Figure 1:**
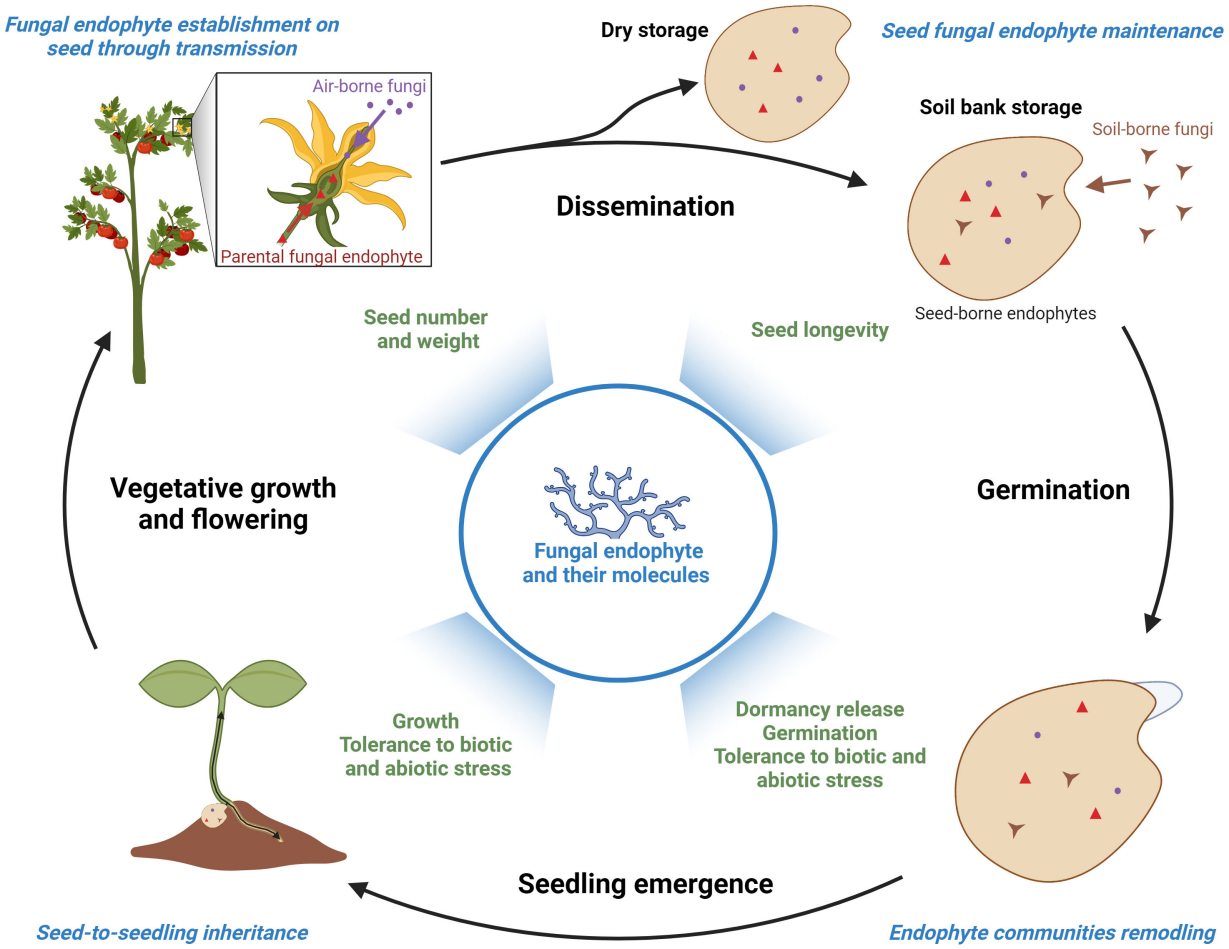
Origins and biological functions of fungal endophytes during seed-to-seed cycle. Seed fungal endophytes can be inherited from the mother plant (red labels) by vertical transmission during the early stages of seed development or acquired from air-borne (purple labels) and soil-borne (brown labels) populations before, during or after seed dispersal. During germination and early seedling development, a portion of the seed-borne endophytes migrate towards the emerging plantlets (vertical transmission). Major stages of endophytic community acquisition, maintenance and dynamics are highlighted in blue. The benefits of endophytes and their bioactive molecules for seed and seedling performance are indicated in green. Created with BioRender.com.

As presented in **Figure 1**, seed endophytic communities are partly inherited from the microbiota of the mother plant via vertical transmission, through asexual (vascular tissues, intercellular spaces) and possibly sexual (gametophytes) routes (Abdelfattah et al., 2022). Endophytic fungi can also be acquired from seed environment by horizontal transmission, from air and rain during seed development and from soil after seed dispersal (U’Ren et al., 2009; Nelson, 2018). In this last case, the seed coat that protects seeds and prevents the penetration of pathogens represents a barrier and a harsh environment for endophyte penetration and survival. Moreover, the low diversity of endophytic fungi observed in seeds compared to other plant tissues may result from interactions among seed-transmitted microorganisms including endophytes so as plant defense mechanisms (Newcombe et al., 2018). After dispersal, endophytic fungal populations further evolve depending on seed conservation. On the one hand, soil seed banks can be infected by soil-borne microbes and an increased fungal diversity is observed over time (Gallery et al., 2007; U’Ren et al., 2009). On the other hand, the viability of fungal endophytes can be reduced during post-harvest storage of dry seeds depending on the storage temperature and humidity (Rolston et al., 1986; Gundel et al., 2009; Lane et al., 2018). A recent study carried out on seeds from wild banana relatives conserved in seed banks also suggests that loss of seed viability correlates with specific modifications of the fungal endophyte community (Hill et al., 2021). Moreover, post-harvest treatments with fungicides (Hill and Brown, 2000; Leyronas et al., 2006) but also insecticides (Nettles et al., 2016; Solanki et al., 2019) reduce seed endophytic fungi populations. When germination occurs, the growth of endophytes is reinitiated and they are mobilized to colonize plantlets (Johnston-Monje et al., 2021). A further reduction of seed-borne endophyte diversity is therefore observed in the seedlings, due to differences in the growth rate among endophytic fungi (Ganley and Newcombe, 2006; Barret et al., 2015). As recently suggested, seed-borne endophytes may subsequently colonize specific organs (root, stem) through selective mechanisms currently unknown (Abdelfattah et al., 2022). Beside their importance for seed biology, seed endophytes could therefore also play critical functions in early plantlet establishment.

## Seed-borne endophytic fungi: a high potential to improve seed performance

Numerous studies have reported beneficial effects of fungal endophytes on different aspects of seed biology, i.e. seed development, germination (including dormancy release) under optimal or stress conditions and longevity (**Table 1** and references therein). These studies have been carried out on both crops, *e.g*. tomato, wheat or soybean, and non-crop, *e.g. Achnatherum inebrians* seeds, using core or flexible endophytic fungi. Although fragmented, they provide elements to understand how endophytic fungi can modify seed physiology and improve seed performance. The best examples come from studies performed on *Epichloe* spp. (anamorph genus *Neotyphodium* spp., family Clavicipitaceae), which are obligate symbionts of cool-season grasses and strictly seed transmitted (Zhang et al., 2010; Ma et al., 2015; Chen et al., 2016; Bao et al., 2019; Ahmad et al., 2020; Chen et al., 2021). They interact mutualistically with their hosts, promoting growth, reproduction and resistance to pests, mainly by producing alkaloids (Kuldau and Bacon, 2008). *Epichloë* infection enhances *Achnatherum inebrians* germination rate under optimal and stress, e.g. extreme temperature, salt stress, extreme pH or heavy metals, conditions (Zhang et al., 2010; Chen et al., 2016; Bao et al., 2019; Ahmad et al., 2020; Chen et al., 2021). It also promotes seed dormancy release (Chen et al., 2021). Dormancy is a critical parameter for seed survival in nature, avoiding seed germination under stress, and homogeneity and germination speed in agriculture. A general mechanism through which *Epichloë*, and other fungal endophytes such as *Penicilium* sp. (Hubbard et al., 2012; Vujanovic et al., 2016; Shearin et al., 2018), *Cladosporium cladosporioides* (Qin et al., 2016) or *Fusarium verticillioides* (Radhakrishnan et al., 2013), promote seed germination and dormancy release in these different contexts is likely the modification of hormonal equilibrium. Indeed, higher and lower content of hormones promoting [gibberellins (GA), auxin] or inhibiting germination [Abscissic acid (ABA)], are respectively measured in endophytic seeds upon imbibition (Radhakrishnan et al., 2013; Vujanovic et al., 2016; Chen et al, 2021). The ABA/GA balance is critical for seed germination and essentially regulated at the transcriptional level (Carrera-Castaño et al., 2020). Endophytic fungi might therefore modulate hormone-related gene expression in seeds, as reported for *Epichloë* in *Achnatherum inebrians* plants (Zhao et al., 2021). Alternatively, *Epichloë* endophytes could interact with hormonal balance through loline alkaloids production, which have been suggested to participate in promoting growth either directly or indirectly via the modulation of hormones, such as polyamines, with which they share precursor amino acids (Schardl et al., 2007). However, no results demonstrate the direct role of alkaloid on seed biology so far. *Epichloë* infection also triggers important modifications of the seed metabolome (Zhang et al, 2019; Liang et al, 2023). Beside the accumulation of alkaloids, changes in the contents of purine and amino acid derivatives, lipids and sugars have been reported (Zhang et al., 2019; Chen et al., 2021; Liang et al., 2023). The comparison of metabolomic and transcriptomic data suggest that *Epichloë* infection affects seed metabolism at least partly through transcriptional regulation (Chen et al., 2016; Rahnama et al., 2023). As previously shown, metabolic resumption is critical for efficient germination and fungal endophytes could participate in this process (Rosental et al., 2014).

A major outcome of seed infection by endophytic fungi is the improvement of seed germination under abiotic stress conditions (Hubbard et al., 2012; Radhakrishnan et al., 2013). In this context, metabolites accumulated in *Epichloë*-infected seeds could participate in a better tolerance to stress at the germination stage, as recently proposed in root and leaves (Hou et al., 2021; Liu et al., 2021). For instance, Li et al. (2020) reported that seeds infected with *Epichloë* accumulated higher contents of proline and soluble sugars when conserved in sub-optimal conditions, leading to a prolonged longevity. In addition, various seed species infected with endophytic fungi present a higher level of antioxidant defense (Zhang et al., 2010; Zhang et al., 2012; Ma et al., 2015; Li et al., 2020; Wang et al., 2020). Reactive oxygen species (ROS) content in seeds is a key factor for seed capacity to germinate (Bailly, 2019). As shown by Li et al. (2020), under unfavorable conditions, seeds infected with *Epichloë* exhibited a higher level of antioxidant activities, e.g. superoxide dismutase, ascorbate peroxidase, correlated with a lower ROS content and limited oxidative damages. Further evidence of ROS scavenging by *Epichloë* is the correlation between loline produced by the fungal endophyte and the production of the antioxidant molecule tocochromanol in *Lolium multiflorum* seeds (Gundel et al., 2018). From the numerous evidences obtained in vegetative organs (reviewed by Chen et al, 2022), it is expected that endophytic fungi modulate seed tolerance to stress through the modification of gene expression. So far, transcriptomic analyses have only been performed in cold-stressed *Achnatherum inebrians* seeds infected with *Epichloë* (Chen et al., 2016). In this context, seed infection impacts the expression of more than 150 genes, including stress response genes involved in protein folding, ROS scavenging and membrane lipid remodeling, that participate in cold tolerance (Chen et al., 2016). A generalization of such approach to multiple seed and endophyte species and stress conditions are now needed to identify common and specific transcriptomic signatures and associate them to stress tolerance at the germination stage.

Although less investigated, seed formation and yield are stimulated by endophytic fungi (Rice et al., 1990). This might reflect the improvement of nutrient translocation from the mother plant to the developing seeds by endophytic fungi. The positive effect of endophytes on seed number and weight is particularly significant for plants exposed to abiotic stresses (Hubbard et al., 2014; Parmar et al., 2022). Strikingly, the infection during seed formation on the mother plant also imprints seed tolerance to stress after release, at the germination stage (Hubbard et al., 2014). The transgenerational transmission of stress tolerance is associated with specific epigenetic regulations. Forte et al., 2023 recently evidenced that *Epichloë* infection triggers specific modifications of *Lolium perenne* DNA methylation. Whether it participates to the maintenance of stress tolerance in seeds over generation is currently unknown but provides a new angle to tackle seed-borne endophytic fungi functions.

Pathogen and feeder attacks are major threats for seed germination and seed endophytic fungi provide protection against a wide range of bio-aggressors (Ma et al., 2015; Li et al., 2017). Interestingly, protection by seed-borne endophytes frequently extends to later development stages following plantlet emergence (Ma et al., 2015). Protective mechanisms include the production of antimicrobial compounds, e.g. alkaloids, terpenoids or cell wall-degrading enzymes, by the endophytes, so as the activation of plant defense mechanisms through the stimulation of plant salicylic or jasmonic acid production (Schmid et al., 2017; Kou et al., 2021). *Epichloë* endophytes have been highlighted for their antifungal activity provided by the constitutive production of antifungal molecules (Niones and Takemoto, 2014; Fernando et al., 2020). Beside *Epichloë*, several seed-borne fungal endophytes, e.g. *Penicillium crustosum*, *Sarocladium zeae*, *Sarocladium strictum* or *Lecanicillium lecanii* have been reported to produce antimicrobial compounds (Valente et al., 2013; Shen et al., 2014; Błaszczyk et al., 2021; Kim et al., 2022). Nevertheless, their role in mitigating pathogen attacks in seeds and their mode of action is still not fully known. Molecules identified in seeds include loline (Justus et al., 1997; Gundel et al., 2018) and ergot alkaloids (Ahimsa-Müller et al., 2007), peramine (Ball et al., 1997a) and lolitrem B (Ball et al., 1997b). Although unknown in seeds, their role can likely be extrapolated from that in vegetative organs. They could participate in plant defense against herbivores as reported for *Epichloë* lolines and ergot alkaloids, indole diterpenoids (lolitrems) and pyrrolopyrazines (peramine) (Bush et al., 1997). The neurotropic activities of lolines, and the activity of peramine as a feeding deterrent, can significantly enhance competitiveness of grasses housing such alkaloid-producing endophytes (Bush et al., 1997). However, loline alkaloids exhibit a broader range and more overt toxicity to insects than peramine (Schardl et al., 2004). Apart from *Epichloë*, the seed endophyte *Undifilum oxytropsis* produces swainsonine, an alkaloid bioactive on neurological functions, and that protects host plant from herbivores (Oldrup et al., 2010; Cook et al., 2011). A major limitation for the use of such endophytes to control feeder attacks is the anti-vertebrate activities of some of their metabolites, i.e. indole diterpene and ergot alkaloids, that are responsible for livestock intoxication (Bacon, 1995). *Epichloë* strains altered in alkaloid production that retain protection potential, with minimal negative effects on livestock, have therefore been selected. These strains produce neither lolitrem B nor ergovaline and the sole production of peramine provides a defense against major pest insects. They are now commercially available and commonly used by farmers to improve pasture performance in agrosystems (Eady, 2021).

## Future developments towards endophyte-based solutions in seed treatments

Recent progress based on metagenomics uncovered the huge diversity of endophytic fungal communities in seeds. This knowledge paves the way for engineering seed endophytic microbiota to improve seed performance, in particular under stress conditions. In this view, endophytic fungal strains selected from tolerant seeds may be used to improve the germination of sensitive seed varieties under stress, a strategy that has been successful for promoting plant growth under stress (Sampangi-Ramaiah et al., 2020). Beyond, endophytic populations from wild or tolerant relatives of selected crops might represent a valuable source to build synthetic communities (SynCom) for seed improvement. Nevertheless, this strategy remains challenging (de Souza et al., 2020). On the one hand, the design of efficient SynComs will require a better understanding of the individual, synergistic and cumulative effects of identified seed-borne endophytes on seed performance. On the other hand, their stability following seed inoculation has to be assessed.

An alternative strategy to the inoculation of seeds with endophytes themselves is the application of bioactive molecules produced by fungal endophytes. In this view, seed-borne endophytes are likely candidates to produce biostimulants active on seed biology. So far, only a handful of seed-borne endophytic fungi have been studied in this respect, and the potential value of their metabolites has essentially been considered for pest and pathogen management. As the benefits of fungal endophytes during seed cycle go far beyond the sole protection against biotic stresses, much gain can be anticipated from the discovery and use of the chemical mediators that underlie such services. The extraction and purification of bioactive molecules require the cultivation of fungal species and a major bottleneck of this approach is the gap between the number of identified seed-borne endophytic fungi and those cultivable. Moreover, culture conditions differ from the natural seed environment and will modify fungal metabolomes. Optimized approaches to isolate and cultivate seed-borne endophytes, so as to purify and test the bioactivity of their metabolic extracts, will therefore be required to identify new and robust seed biostimulants. Aside from providing potential solutions for agriculture, the study of the mode of action of these extracts will bring important information on the regulation of seed development, germination and/or longevity.

## Author contributions

FR: Writing – original draft, Writing – review & editing. CK: Writing – review & editing. KC: Writing – original draft, Writing – review & editing. CD: Writing – review & editing. SP: Writing – original draft, Writing – review & editing. CB: Writing – review & editing. EB: Writing – original draft, Writing – review & editing.
